# Development of a qPCR molecular diagnostic assay for the detection of kiwi *Eimeria* species and its application to determine tissue-specificity of species causing coccidiosis in North Island brown kiwi (*Apteryx mantelli*)

**DOI:** 10.1007/s00436-025-08521-0

**Published:** 2025-07-04

**Authors:** Emma Scheltema, Kerri Morgan, Stuart Hunter, John Mackay, Preet Singh, Laryssa Howe

**Affiliations:** 1 School of Veterinary Sciences, Massey University, Palmerston North, New Zealand; 2 Wildbase, Massey University, Palmerston North, New Zealand; 3 Dnature Diagnostics and Research Ltd., Gisborne, New Zealand

**Keywords:** *Eimeria*, Extraintestinal infection, qPCR, Coccidiosis, CO1

## Abstract

Juvenile kiwi (*Apteryx* spp.) within captive-rearing programmes commonly suffer from coccidiosis, which primarily affects the intestine but can also impact other organs, such as the kidneys, liver, lung, and spleen (Morgan  et al. Avian Pathol 42:137–146 2013). In some immune-compromised birds, disease causes significant morbidity and, occasionally, mortality (Morgan et al. NZVJ 62:315–320 2014); however, understanding of the biology of disease-causing *Eimeria* species in kiwi is limited. A probe-based qPCR assay targeting a 115-bp fragment of the *Eimeria* mitochondrial cytochrome c oxidase I (CO1) gene was developed to identify three distinct kiwi *Eimeria* species: the two species most commonly recovered from faeces, *Eimeria kiwii* and *Eimeria apteryxii*, as well as the newly described species, *Eimeria koka* (Scheltema et al. Syst Parasitol 102:30 2025). The qPCR assay was then applied to retrospectively analyse formalin-fixed paraffin-embedded intestine, kidney, liver, lung, and spleen tissues from ten historic post-mortem cases from kiwi diagnosed with extraintestinal coccidiosis. This novel assay detected infection more often (33/47 tissues) than manual histopathological identification (25/47 tissues). Only one species, *E. koka*, was detected in extraintestinal tissues with the highest prevalence (9/10) in kidney tissues. In contrast, *E. kiwii* was reliably detected in 8/9 intestinal tissues but was not detected in the other tissues tested. *E. apteryxii* was not detected in any of the tissues analysed. These findings suggest that kiwi are infected by at least one intestinal and one renal-specific species, the latter of which is suspected to disseminate under certain conditions to other organs of the body.

**Supplementary Information:**

The online version contains supplementary material available at 10.1007/s00436-025-08521-0.

## Introduction

Coccidiosis, a disease caused by endogenous intracellular infection with protozoan parasitic species of the genus *Eimeria*, is one of the most frequently described parasitic diseases in kiwi and occurs routinely in captive- and creche-reared kiwi chicks in intensive conservation captive-rearing programmes, such as Operation Nest Egg (ONE), as well as occasionally in immunocompromised adults (Morgan et al. 2014). At least six *Eimeria* species have been identified and morphologically described from kiwi faecal samples (Morgan et al. 2017; Coker et al. 2023). The most commonly observed species, *Eimeria kiwii* Morgan et al. 2017, and *Eimeria apteryxii* et al. 2017, are routinely detected in kiwi faeces in significant numbers (Bassett 2012; Morgan et al. 2017). However, faecal oocyst counts, which typically consist of multiple species, often do not correlate with observed clinical symptoms in infected birds, possibly due to variation in the pathogenicity of different *Eimeria* species. Like most *Eimeria* species, the primary infection site in kiwi is the tissues of the gastrointestinal tract; however, less commonly, infection has also been recorded extraintestinally, usually in the liver and kidney, and, in a few cases, the lung and spleen, primarily in juvenile birds (Morgan et al. 2012, 2013).

Eimeriid coccidians have traditionally been thought to be highly host-, tissue-, and site-specific (Marquardt 1976; Ball et al. 1989; Kogut 1990). Experimental work in chickens and rats has proven that the motile infective stages, or sporozoites, of *Eimeria* species have the ability to migrate in the body to very specific sites of infection and that site-specificity is very strongly predetermined (Ball et al. 1989). While the intestine is the most common site of coccidia infection across a range of vertebrate host species, there are *Eimeria* species that naturally infect other organs of the body. In avian hosts, there are a number of species that infect the renal tissues, for example, *Eimeria truncata* in geese (*Anser anser domesticus*) (Gajadhar et al. 1983), *Eimeria boschadis* in mallard ducks (*Anas platyrhynchos*) (Ball et al. 1989), *Eimeria fraterculae* in Atlantic puffins (*Fratercula arctica*) (Leighton & Gajadhar 1986), and *Eimeria wobeseri* sp. n. in herring gulls (*Larus argentatus*) (Gajadhar & Leighton 1988). Other species infect the liver, such as *Eimeria steidae* in rabbits (*Oryctolagus cuniculus*) (Pellérdy 1974) and *Eimeria debliecki* in pigs (*Suis* spp.) (Desser 1978).

In contrast, disseminated forms of coccidiosis—where a species occurs outside its normal development location, becoming widely distributed through various tissues of the body—are less common but have also been described in several host species (Novilla et al. 1981; Dubey et al. 2019). Disseminated coccidiosis has been well described in several species of birds, such as the crane (*Grus* spp.), where it causes widespread infection of viscera, and in corncrakes (*Crex crex*), where it has been detected in the spleen and liver (Serna et al. 2018). It is thought to be more common when the host’s immunity is reduced, when individuals are stressed and/or placed in a highly contaminated environment (O’Brien et al. 2011)—especially juveniles whose immune system is still under development (Long 1970; Serna et al. 2018; Lillehoj et al. 2019). Therefore, while *Eimeria* species generally migrate to infect specific locations within the body, under certain conditions, the ecological niche of some species may become more fluid.

In conservation-managed rare or threatened wildlife species, investigation of internal lifecycle stages of endogenous parasites is limited by the inability to carry out experimental infection studies. Therefore, knowledge of parasite biology and pathogenicity is often elucidated through the non-invasive study of faecal material and retrospective analysis of preserved post-mortem tissues. Morgan et al. (2012, 2013) conducted in-depth analyses of post-mortem histopathological samples from infected kiwi and characterised the endogenous morphology of intracellular forms of *Eimeria* species in the intestinal and extraintestinal tissues. This work identified at least three different *Eimeria* morphotypes infecting various sections of the intestine, suggestive of different species, and documented a distinct morphological difference in the sexual stages of coccidia observed in renal tissues likely to represent a species distinct to those observed in the intestine. Due to a lack of observed sexual lifecycle stages in the hepatic tissues, the species infecting the liver was suggested to complete some stages of the lifecycle in the intestine. Further establishing which species of *Eimeria* infect each tissue type within the kiwi is challenging as the majority of coccidial infections are of mixed species, and the morphology of intracellular infective stages within each organ cannot be reliably matched to the morphology of exogenous oocysts recovered in faeces, upon which species are differentiated.

Recently, molecular analysis of tissues from a kiwi that died due to renal and intestinal coccidiosis identified a single species in infected kidney and lung tissue that was a match for a newly described species, *Eimeria koka* (Scheltema et al. 2025). However, the prevalence of this species, and other kiwi *Eimeria* spp., within kiwi tissues is unknown. Thus, in this retrospective study, a real-time probe-based quantitative PCR (qPCR) assay was developed to identify several species of kiwi *Eimeria*. This assay was then applied to test a selection of preserved tissues (intestine, kidney, liver, spleen, and lungs) from ten historic post-mortem cases of birds identified to have extraintestinal coccidiosis at necropsy from 2012 to 2021. These were analysed molecularly using a generic *Eimeria* species qPCR assay to identify initially whether infection was present and then—using hydrolysis probes designed to target *E. kiwii*, *E. apteryxii*, and *E. koka*—to determine if any of these species were present in each tissue type.

The aim of this study was to determine the site-specificity and tissue distribution of different kiwi *Eimeria* spp. and thus gain a greater understanding of their infection dynamics in kiwi. This information will assist in developing a better understanding of kiwi *Eimeria* species life cycles and provide background information for future work linking *Eimeria* species tissue infectivity to clinical symptoms and development of strategies to treat different forms of disease.

## Materials and methods

### qPCR assay development

#### Assay controls

A genomic DNA panel of seven samples representing four species of kiwi *Eimeria*: three target (*E. kiwii*, *E. apteryxii*, and *E. koka*), one non-target (*Eimeria paraurii* Morgan et al. 2017) species, and a related coccidia, *Isospora* spp., recovered from kiwi faeces were used to test assay specificity. Faecal samples from captive-reared North Island brown kiwi (*Apteryx mantelli* Bartlett, 1852) and rowi (*Apteryx rowi* Tennyson et al., 2003) juveniles, aged between 1 and 6 months old, were obtained through routine diagnostic screening carried out at Massey University Parasitology Laboratory and consisted of both single- and mixed-species infections. Coccidia were morphologically identified to species-level microscopically via Mini-FLOTAC analysis (Coker et al. 2020). As multiple species are usually present in faeces from infected kiwi, individual oocysts of *E. kiwii* and *E. apteryxii* were also collected for DNA extraction using a 3-axis hydraulic micromanipulator (Narishige coarse (MN-4) and fine (MMO-4) micromanipulator with Narishige IM112 Pneumatic microinjector) in groups of five to 20 morphologically identical oocysts eluted into 30μL sterile PBS (as per Yang et al. 2015), in an attempt to generate single-species DNA sequences.

One infected hepatic tissue sample obtained from a 7-month-old captive juvenile North Island brown kiwi (*A. mantelli*) from the Massey University School of Veterinary Science’s pathology collection, which had been previously molecularly determined to contain *E. koka*, was also included.

#### DNA extraction

Faecal samples and oocysts in 0.5–1 mL distilled water were frozen at − 80 °C for > 24 h. DNA was extracted from 0.15 g faeces, or 200μL oocyst solution, using the Quick-DNA Fecal/Soil Microbe DNA Miniprep extraction kit (ZYMO Research, Orange County, CA, USA), following manufacturer’s instructions with modifications described by Coker (2021). DNA was eluted into 70μL elution buffer. A water extraction negative control was used in each extraction group. Extracted DNA was tested on a Nanodrop™ 2000 spectrophotometer (Thermo Fisher Scientific, Waltham, MA, USA) to measure DNA concentration and quality and stored at − 20 °C until analysis.

FFPE samples containing a single tissue type were prepared for extraction using a microtome with a fresh blade between samples, for PCR analysis (10 µm) flanked by two thinner samples (4 µm) for H&E staining. DNA extraction was performed on 10 µm scrolls of FFPE tissues following the manufacturer’s guidelines for a commercially available kit (Qiagen DNeasy® Blood and Tissue kit, Valencia, CA, USA). DNA was eluted using only one elution of 70 µL of the provided elution buffer. Extracted DNA was quantified and stored as for faecal and oocyst samples as described above.

#### Molecular description of control samples

Mitochondrial cytochrome *c* oxidase I gene (CO1) sequence information from DNA extracted from coccidia-infected kiwi extraintestinal FFPE tissues and *Eimeria* spp.-positive kiwi faeces was compiled. Briefly, conventional PCR was carried out targeting a 465-bp region of the *Eimeria* CO1 gene using CO1F2/CO1R2 primers developed by Yang et al. (2013) (Table 1). If no amplification was observed due to poor quality or fragmented DNA, a smaller 220 bp region was targeted using CokerF2/CO1R2 primers developed by Coker (2021) (Table 1). The PCR reaction conditions for CO1F2/CO1R2 were as follows: 1X PCR buffer, MgCl_2_, 200 nM dNTPs (Invitrogen, Waltham, MA, USA), 500 nM each forward and reverse primers, 5U Platinum™ Taq DNA polymerase (Invitrogen), and up to 50 ng DNA for a total reaction volume of 25 µL. Cycling conditions were the following: initial denaturation at 96 °C for 5 min, then 35 cycles of denaturation at 94 °C/20 s, annealing at 52 °C/30 s, extension at 72 °C/90 s, with a final extension at 72 °C for 10 min.

**Table 1:** Primers and probes for amplification and fluorescent detection of partial mitochondrial cytochrome c oxidase (CO1) genes from kiwi *Eimeria* spp

Primer/probe name	Sequence (5′−3′)		Target sequence length (bp)	Target	Reference
CO1F2	5′ TAA GTA CAT CCC TAA TGT C 3′	465		*Eimeria* spp.	Yang et al. (2013)
CO1R2	5′ GTC ATC ATA TGR TGT GCC CA 3′				
CokerF2	5′ AYG ATG CYT CYT TTA ATG GTG A 3′	220		*Eimeria* spp.	Coker (2021)
CO1R2	5′ GTC ATC ATA TGR TGT GCC CA 3′				Yang et al. (2013)
*Eimeria* TM-F	5′ CAT YTA TTC TGG TTC TTT GGA CAC 3′	115		*Eimeria* spp.	*This paper*
*Eimeria* TM-R1	5′ GAW GGA CCT CCA AAS ACT RAT TTA 3′				
EA-560	5′ TGC GGA AGT AGA CAG TGT TT 3′ (CAL Fluor Orange 560, BHQ-1 plus™)	-		*Eimeria apteryxii*, GenBank PQ609358	*This paper*
EK-FAM	5′ TGC TGA TGT AGA TAA AAT TTG AGA T 3′ (FAM, BHQ-1 plus™)	-		*Eimeria kiwii*, GenBank PQ609359	*This paper*
Tissue-FAM	5′ TGC TGA GGT AGA TAG TGT CT 3′ (FAM, BHQ-1 plus™)	-		*Eimeria koka*, GenBank PQ609361	*This paper*

For CokerF2/CO1R2, the conditions were as described by Coker (2021). Briefly, 1X PCR buffer, 1.5 mM MgCl_2_, 200 nM each of dNTPs (Invitrogen), forward and reverse primers, 5U Platinum™ Taq DNA polymerase (Invitrogen), and up to 45 ng DNA were combined for a total reaction volume of 20 µL. Cycling conditions were the following: initial denaturation 94 °C for two min, then 40 cycles of denaturation at 94 °C/20 s, annealing at 55 °C/30 s, extension at 72 °C/30 s, with a final extension of 72 °C for 10 min.

PCR products were visualised on 1.5% w/v agarose gels (UltraPure Agarose, Invitrogen) using RedSafe Nucleic Acid Staining Solution (iNtRON Biotechnology, Gyeonggi-do, South Korea) (see Online Resource 1). Samples with bands of the correct size (220 or 465 bp) were purified for sequencing. The positive PCR products of FFPE tissue samples were purified using PureLink Quick PCR purification kit (Invitrogen), whereas the PCR products from faecal DNA and individual oocyst extractions were excised from the agarose gel and frozen overnight at − 20 °C before using a homemade spin-column protocol (Sun et al. 2012). All purified products were sent for Sanger sequencing (forward and reverse) at Massey Genome Service (Massey University, Palmerston North, New Zealand).

**Fig. 1:**
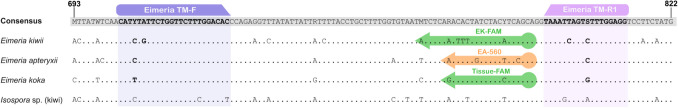
Alignment of primers (*Eimeria* TM-F and TM-R1) and species-specific hydrolysis probes (EK-FAM, EA-560 and Tissue-FAM) designed for the present study, with kiwi *Eimeria* spp. CO1 consensus sequence, *Eimeria* spp. specific reference sequences and *Isospora* sp.(GenBank PQ609360) isolated from kiwi faeces. Numbers at each end of the sequence indicate base position on *Eimeria* CO1 gene

Sequences were analysed in Geneious Prime v. 11.0.14.1 + 1 (Biomatters, Auckland, New Zealand), and any with a quality score of < 50% were omitted from further analysis. Sequences from samples that were morphologically identified as *E. kiwii* (*n* = 3) and *E. apteryxii (n* = 3), respectively, as well as a set of identical sequences extracted from extraintestinal tissue samples, previously confirmed as synonymous with *E. koka* (Scheltema et al. 2025) (renal, hepatic, and pulmonary, *n* = 9), were submitted to NCBI BLAST to confirm them as unique *Eimeria* species. The sequences were then aligned in Geneious Prime (Biomatters) to generate consensus sequences for each of the three species (*E. kiwii*,* E. apteryxii*, and *E. koka*), which were used for primer and probe design (Fig. 1). Unique sequences were submitted to the NCBI GenBank database (*E. apteryxii*—PQ609358; *E. kiwii*—PQ609359; *E. koka*—PQ609361; *Isospora* sp. (ex. kiwi)—PQ609360).

### Primer and probe design

Generic *Eimeria* primers were designed by eye based on consensus sequences generated for a conserved 115-bp fragment of the mitochondrial cytochrome *c* oxidase I gene of the three target species (Table 1).

The generic primer combination (*Eimeria*-Tm-F/*Eimeria*-Tm-R1) was run across the panel of representative species DNA samples (*E. kiwii*, *E. apteryxii*, *E. koka*, *E. paraurii*, and *Isospora* sp. recovered from kiwi faecal samples) to assess the specificity of each primer pair/combination with the goal of amplifying all kiwi *Eimeria* spp. and excluding the *Isospora* spp. (which have not been confirmed as parasites of kiwi to date)*.* The generic *Eimeria* amplification reaction was carried out containing 1 × BioRad SYBR Green Supermix, 200 nM each of forward and reverse primers, and up to 45 ng DNA in a total reaction volume of 20 µL. All qPCR assays were performed on the Mic qPCR cycler (Biomolecular Systems, Australia). Cycling conditions for the SYBR green assay were the following: initial denaturation at 95 °C for 2 min, then 40 cycles of denaturation at 95 °C/10 s, annealing at 55 °C/30 s and extension at 72° C/30 s. Melting curve analysis was included (65–85 °C at 0.5 °C increments and 0.1 °C/s)*.* The fluorescence detection threshold was set to ignore cycles below 15. A water negative was used as a control in each run.

Three fluorescent probes were also designed to specifically bind to each of the three target *Eimeria* species (*E. kiwii*, *E. apteryxii*, and *E. koka)* (Table 1). The probes utilised the BHQplus technology (LGC Biosearch Technologies, Petaluma, CA, USA) so that shorter (more specific) probes of appropriate Tm could be used in the assays. The quality parameters (hairpins, complementarity) of each primer and probe were evaluated in Geneious using Primer 3 plug-in (Koressaar and Remm 2007; Untergasser et al. 2012). Each probe was trialled with the *Eimeria*-Tm-F/*Eimeria*-Tm-R1 primer set with the same panel of assay control samples to test for binding specificity to ensure that the known samples were correctly identified and that samples of the non-target species were not detected. Each species-detection reaction contained 1 × PerfeCTa qPCR Toughmix (Quantabio, Beverly, MA, USA), 400 nM each forward and reverse primer, 200 nM of species-specific probe (LGC Biosearch Technologies (Petaluma, CA, USA)), and up to 45 ng faecal DNA in a total reaction volume of 20 µL. Each probe was run in a single reaction and not multiplexed. Cycling conditions on the Mic qPCR cycler for the probe-based species identification protocol were the following: initial denaturation 95 °C/2 min, then 38 cycles of denaturation 95 °C/5 s, and annealing at 60° C/20 s. Fluorescence was detected on FAM (green channel) or Cal-Fluor-Orange 560 (yellow) channels, depending on the probe. The fluorescence detection threshold was set to 0.5, ignoring cycles below 15. Samples were considered positive for that species if resulting Cq values were between 19 and 37 cycles. A water negative was used in each run.

### Brown kiwi retrospective tissue cases

#### Case selection

Formalin-fixed paraffin-embedded (FFPE) tissue blocks from ten North Island brown kiwi (*Apteryx mantelli*), which had previously been diagnosed with extraintestinal coccidiosis via histopathological examination, were collected from the Massey University School of Veterinary Science’s pathology collection for retrospective analysis. Samples from the liver, kidney, spleen, lungs, and intestine were targeted from all birds where available.

#### Demographics

The analysed tissue samples were all from young kiwi (chicks to juveniles), aged between 8 days and 7 months old. All had been living in captive-rearing facilities or creche sites (see Online Resource 2).

#### DNA extraction

DNA was extracted from FFPE liver, kidney, spleen, lung, and intestine tissues, as described above, for each of the ten cases.

#### Histology

Histological assessment was carried out on new haematoxylin and eosin (H&E)-stained slides prepared from archived formalin-fixed paraffin-embedded (FFPE) tissues, as described above. Both H&E slides taken on either side of the FFPE sample for DNA extraction were thoroughly examined by microscope at × 100–400 magnification for the presence of intracellular coccidial stages, and the presence of asexual or sexual stages was recorded.

#### Sample analysis using probe-based qPCR assay

Extracted FFPE tissues were screened via qPCR amplification, initially using the generic kiwi *Eimeria* assay (primers *Eimeria*-Tm-F and *Eimeria*-TM-R1 and SYBR Green detection) and then in combination with species-specific probes as described above.

In order not to overwhelm the assay with nonspecific DNA from mixed genomes present in tissue, samples were initially screened using the *Eimeria* generic assay at the standard concentration of 40 ng/reaction across all samples. If negative at that concentration, the samples were retested at 80–100 ng/reaction to confirm true negatives. Samples that were *Eimeria* positive at 40 ng/reaction were not retested. All samples, both *Eimeria* spp.-positive and -negative, were then screened using each of the three species-specific probes at 40 ng/reaction, as described above. DNA extracted from both kiwi *Eimeria*-positive (mixed species) and negative faeces and a water negative were used as controls in each run.

#### DNA sequences

A selection of 15 positive DNA samples from case study extraintestinal tissues (hepatic *n* = 5; renal *n* = 7; lung *n* = 1; spleen *n* = 1), at least one from each case study, was amplified using CokerF2/CO1R2 primers (Coker 2021), purified, and sent for Sanger sequencing (both directions) at Massey Genome Service as described above and compared to existing sequences to check similarity and confirm probe specificity.

## Results

### qPCR assay development

**Fig. 2:**
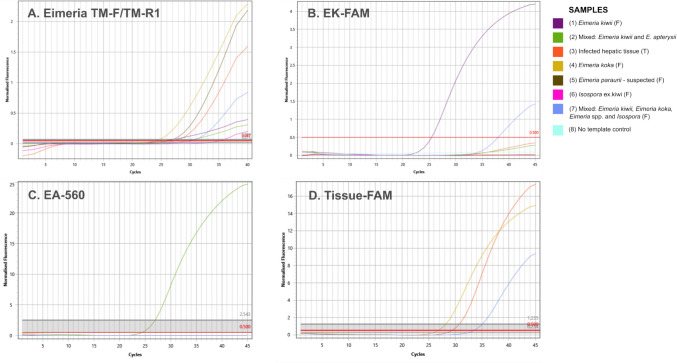
qPCR amplification curves from qPCR screening of representative *Eimeria* DNA samples (*n* = 7) across all assays. **A** *Eimeria* generic assay; **B** *Eimeria kiwii* assay; **C** *Eimeria apteryxii* assay and **D** *Eimeria koka* assay. Sample key indicates if DNA is of faecal (F) or tissue (T) origin and, if faecal, whether it contains a single or multiple species

All samples used as assay controls yielded a detectable product on the *Eimeria* generic assay, including *Isospora* sp. recovered from kiwi (Fig. 2A). EK-FAM probe detected 2/3 panel samples visually identified as containing *E. kiwii.* The third sample, which was identified as a mix of *E. kiwii* and *E. apteryxii*, had low amplification that did not meet the detection threshold (Fig. 2B). Both EA-560 and Tissue-FAM probes successfully amplified samples containing their target species, *E. apteryxii* and *E. koka*, respectively. None of the probes indicated positive amplification for samples containing non-target species, including closely related *E. paraurii* and *Isospora* species recovered from kiwi, demonstrating that the probes were not only genus-specific but also species-specific. The *Eimeria* generic and EK-FAM assays had noticeably lower overall fluorescence than EA-560 and Tissue-FAM assays. Inclusion of DNA extracted from both tissue and faecal samples demonstrated that both *Eimeria* generic and Tissue-FAM assays could successfully detect *Eimeria* in both faecal and tissue samples (Fig. 2A, D).

### FFPE sample analysis

#### Histological examination

Overall, 18/50 (36%) tissues examined in this study were recorded as coccidia-positive on histological examination at the time of the initial necropsy: 9/10 enteric tissues, 2/10 hepatic tissues, 6/10 renal tissues, 1/10 pulmonary tissue, and 1/10 spleen tissue (Table 2).

**Fig. 3:**
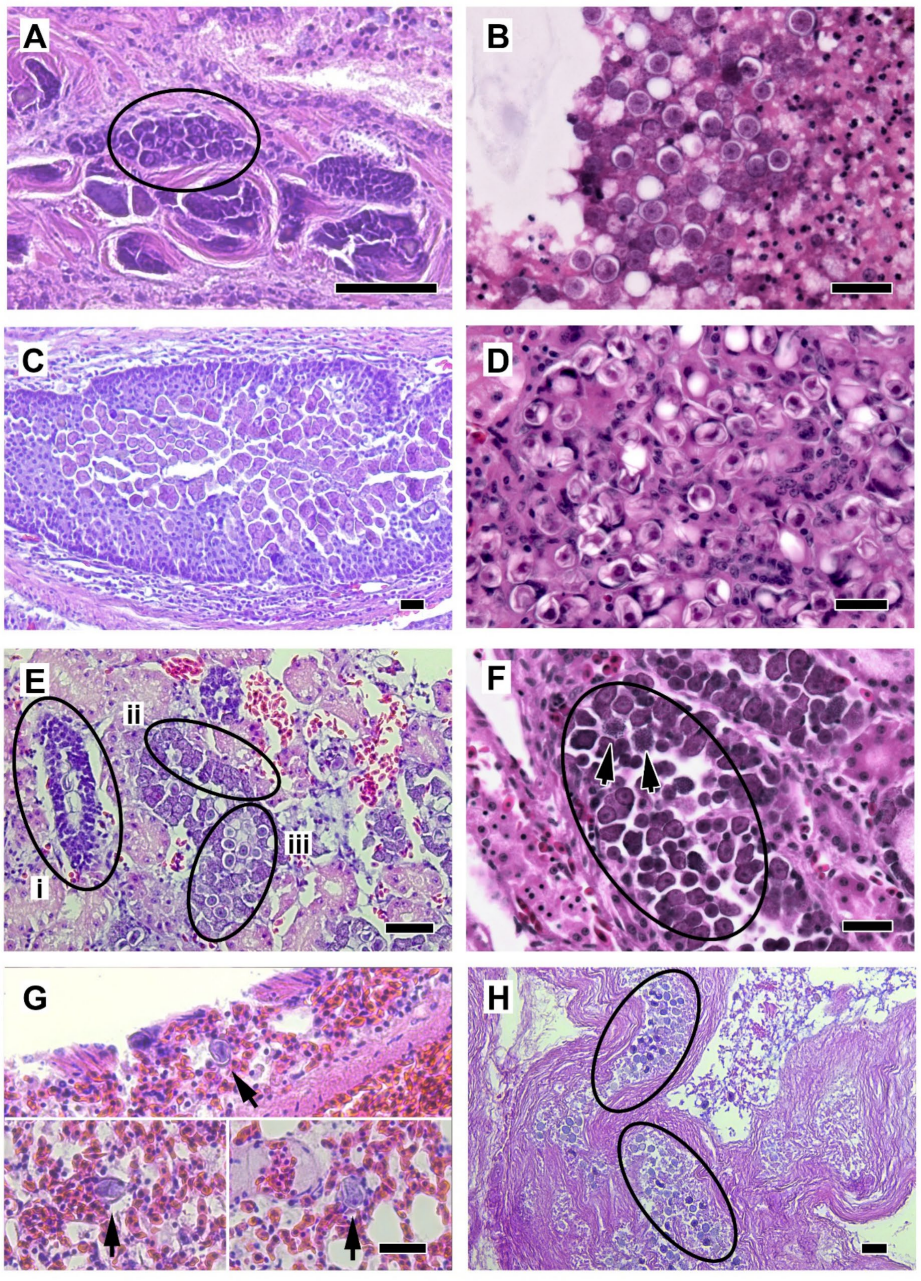
Examples of intracellular coccidia stages from North Island brown kiwi (*Apteryx mantelli*) observed on histological examination of H&E slides in this study. **A** intestinal tissue with coccidial meronts within crypt epithelial cells (circled) (PM accession # 51506); **B** intestinal tissue with extracellular coccidial oocysts admixed with sloughed autolysed mucosa (58596); **C** coccidia meronts within epithelial cells of branches of the ureter (51506); **D** early oval-shaped oocyst stages within epithelial cells of collecting ducts of the kidney (peripheral cortex) (arrow heads) (51506); **E** kidney tissue with arrows indicating (i) immature coccidial meronts, (ii) gametocytes and (iii) formation of early oocysts in the epithelial cells of collecting ducts (49058); **F** kidney tissue with coccidial meronts within collecting tubules (circled)—arrows indicating formation of merozoites within meronts (51484); **G** individual oocysts (arrow heads) observed scattered within the lung interstitium in a single case (49058); and **H** oocysts observed forming in the lumen of the gall bladder of a single case (circled) (51506), with indeterminate origins. Scale bars = 50 µm

Of these tissues, a total of 47 tissue blocks were available for further analysis in this study. Three of the cases had one missing tissue, which could not be located for further analysis. Upon examination of the freshly prepared slides in the present study, both asexual and sexual coccidia stages were observed in the majority of slides prepared from intestinal (8/9) and renal (9/10) tissues (Fig. 3). Sexual stages (oocysts) were also observed in one lung sample. In this case, several circular coccidia oocysts were observed scattered within the lung interstitium and did not appear to be infecting cells of the lung tissue (Fig. 3G). Otherwise, only asexual coccidia stages were observed infecting the cells of the liver, lungs, and spleen (Fig. 3). There were two cases where the original post-mortem examination reported coccidia, but none was visualised on the new slides from that case (Table 2).

### Molecular analysis using probe-based qPCR assay

#### Eimeria generic primers

*Eimeria* spp. DNA was detected using qPCR in at least one tissue of all case studies analysed (32/47 [68.1%] samples) using the generic *Eimeria* primers. *Eimeria* DNA was detected in 8/9 enteric tissues, 6/10 hepatic tissues, 9/10 renal tissues, 7/9 lung tissues, and 1/9 spleen tissues (Table 2).

There was only one tissue sample (50621–hepatic) that was identified as coccidia-positive on initial post-mortem and subsequently found to be negative on repeat histological examination in this study and subsequent molecular testing.

#### Probe assay

Probes specific for three species of kiwi *Eimeria* were used to analyse DNA samples extracted from FFPE pathology samples (Table 2). *E. kiwii* was the most prevalent species (7/9; 77.8%) in enteric tissues of analysed samples, and this species was also detected in one lung sample (58596). It was not detected in any of the hepatic, renal, or spleen samples.

*E. koka* was not detected in any of the enteric tissue samples. However, it was detected in all extraintestinal tissues: 2/10 hepatic samples, 9/10 renal samples, 5/9 pulmonary samples, and 1/9 spleen samples at 40 ng/reaction. A selection of samples from all tissue types that were weakly positive at 40 ng/reaction were run again with the tissue probe at 100 ng/reaction, and there was an increase in detection rates in hepatic tissue only, resulting in two additional positives (total = 4/10)*.*

*E. apteryxii* was not detected in any of the tissues analysed for these ten case studies. Repeat testing of a selection of samples at 150 ng/reaction showed no change in results.

#### Comparison of histological and molecular results

Molecular detection of infection in all extra-intestinal tissues, except spleen, was higher than both the original post-mortem report and the new histological screening carried out in this study (Table 2). Fifteen samples were identified as *Eimeria* positive via molecular analysis despite being histologically negative at the original post-mortem examination. Of these cases, coccidia infection was visually identified on new histopathological slides in half of these cases (7/15). No coccidia were visually observed in the other half despite positive molecular detection (Table 2).

Sexual coccidia stages were only consistently observed in intestinal and kidney tissues, but there were two cases where early oocysts or gametocytes were observed associated outside of these tissues. In one case (51506-hepatic), early-stage circular oocysts were observed on new slides, but both the original post-mortem report and *Eimeria* PCR were negative for coccidia. The observed oocysts were present in small portions of gall bladder tissue, which had inadvertently been included in the liver FFPE block (Fig. 3F). Due to autolysis, it was unclear whether the oocysts originated from epithelial cells lining the bile duct lumen or whether they were, in fact, present within the lumen of the bile duct. Sexual stages were also observed associated with a lung tissue sample from case 49058, and this sample tested positive via qPCR probe assay for *E. koka*.

Six tissue samples were found to be positive for *Eimeria* DNA, but none of the species-specific probes detected amplification (intestinal *n* = 1, hepatic *n* = 3, lung *n* = 1;and spleen *n* = 1). In one of these cases (59312), coccidia were not reported on initial histological examination of the lungs, but they were observed on repeat histological examination in this study. In another case (50621), although splenic coccidia were described in the original histology report, none was observed histologically in this study (Table 2).

#### DNA sequencing

Fourteen extraintestinal tissue samples underwent DNA sequencing. Of these, only nine sequences (renal *n* = 5; hepatic *n* = 2; lung *n* = 1; spleen *n* = 1) were of high enough quality (> 50%) for further analysis. Comparison of sequences showed 7/9 were consistent with the previously identified sequence for *E. koka (*NCBI: PQ283206*)*. The electropherograms from the liver and spleen from case 50621 displayed distinctive overlapped peaks, indicating that there were more than one *Eimeria* species present within each sample.

**Table 2:**
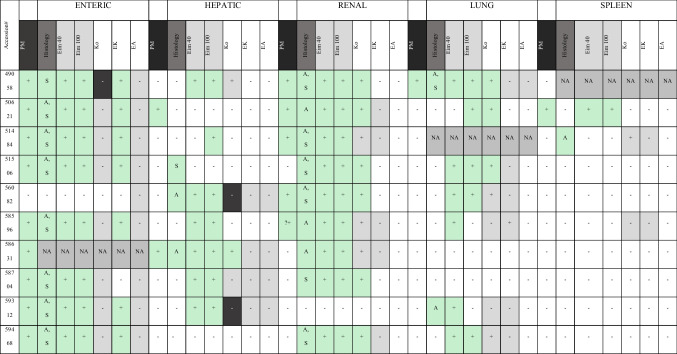
Results of histological and molecular analysis of enteric, hepatic, renal, lung, and spleen tissue from ten North Island brown (Apteryx mantelli) kiwi diagnosed with coccidiosis on post-mortem. The diagnoses from the original postmortem (PM) are reported, and results of histological examination in this study, including the presence of asexual (A) and sexual (S) stages

Each tissue was tested with generic *Eimeria* primers at both 40 ng/rxn and 100 ng/rxn and then tested with each of three species-specific probes at 40 ng/rxn. Note: Samples with probe test results in shaded boxes were retested at 100 ng/rxn, and if the result was different from the 40 ng/rxn result, they are shaded in dark grey (Eim40 = *Eimeria* generic assay at 40 ng/rxn; Eim100 = *Eimeria* generic assay at 100 ng/rxn, *Ko* = *Eimeria koka* probe assay, EK = *Eimeria kiwii* probe assay, *EA* = *Eimeria apteryxii* probe assay) (NA = original tissues not able to be located for testing;? + possible coccidia stages observed on post-mortem)

## Discussion

This study demonstrates the value of molecular tools in elucidating infection patterns in an endogenous intracellular parasitic infection in a threatened wildlife host species. In a species that hosts at least six species of *Eimeria* (Morgan et al. 2017; Coker et al. 2023; Scheltema et al. 2025), species differentiation can be challenging, and without the ability to carry out experimental infection in these threatened avifauna, intracellular infection patterns can be impossible to link to the exogenous oocyst parasitic form. This study demonstrates a valid proof-of-concept for the detection of different species of kiwi *Eimeria* from kiwi tissue samples. While the samples do not represent the pathology of coccidiosis in the wider kiwi population, the findings suggest some possible emerging patterns of infection.

In these ten case studies, coccidia were detected at a higher rate in extraintestinal tissues via molecular methods than via initial post-mortem or visual inspection of new histopathology slides. Due to inconsistent distribution of infection throughout affected organs, it is probable that different sections of tissue analysed molecularly may have infection that differed from that captured in the original post-mortem histology. However, despite screening new H&E slides prepared from either side of the FFPE tissue for extraction, some tissue sections without any identifiable infection on histology were positive via molecular analysis (7/50). The detection of DNA is not necessarily representative of an active infection at the time of death. A higher parasite detection rate via PCR compared to histology could be the result of detection of extracellular forms of infection or, possibly, tissue contamination. In the early stages of infection, circulating infected blood cells and/or sporozoites could be present with no observable intracellular infection or developmental stages on histopathology, as has been documented previously in infections with *Leucocytozoon* spp., *Plasmodium* spp. (Valkiūnas et al. 2009), and *Toxoplasma* spp. (Esteban-Redondo and Innes 1998; Garcia et al. 2006; Carossino et al. 2021). Furthermore, the method of sample preservation, which includes storage of multiple tissues, including intestinal content, together in formalin jars prior to preparation of paraffin-embedded tissues, may have resulted in DNA contamination of the analysed tissues. In this study, it is possible that this may have been the source of small numbers of sexual coccidia stages observed on microscopy of a single lung sample where intracellular infection of the lung tissue could not be confirmed, and possibly also the positive molecular detections of *Eimeria* spp. in other lung tissue samples, including one sample that was positive for *E. kiwii*, a species which was only otherwise detected in intestinal tissues. The presence of varying species, and tissues where no coccidia were detected via PCR, across different organs from the same bird, suggests that if contamination is the source of these differences, it likely makes up only a small number of overall detections. However, greater confidence in ascertaining patterns of infection should be taken from samples where coccidia are detected on both histology and PCR. Future studies would benefit from analysing freshly sampled tissues that have not been through any preservation processes to confirm the findings of this study.

*E. kiwii* was the only one of the three species tested that was detected in the intestinal tract of the kiwi and was not detected in any of the extraintestinal organs, except as suspected contamination. These cases provide no evidence that *E. kiwii* migrates extraintestinally. This finding concurs with previous research by Morgan et al. (2012, 2017) who found that excreted oocysts of *E. kiwii* were morphologically similar to gametocytes and early oocysts (Fig. 3B) observed in intestinal epithelial cells from brown kiwi, which were morphologically distinct from gametocytes observed in other tissues.

*Eimeria apteryxii* is a species commonly recovered from kiwi samples, often in high numbers (Morgan et al. 2017), but, interestingly, was not detected in any of the tissue samples analysed in this study. As the EA-560 probe successfully detected DNA amplification from faecal samples containing this species, both in samples identified as single and mixed species, it seems unlikely that the probe is erroneously binding to another, non-target *Eimeria* species that has been incorrectly identified via sequencing. It is possible that the genome sequence of intracellular forms of the parasite for this species is slightly different to the exogenous oocyst form to which the probe is designed. Genomic variation between different life stages has been documented in studies of other *Eimeria* species (Ng et al. 2002; Katrib et al. 2012) and may have resulted in a sequence mismatch between the designed primers/probe and *Eimeria* DNA extracted from infected organs.

Alternatively, because the presence or absence of *Eimeria apteryxii* in faecal samples from these historical post-mortem cases was unable to be confirmed, it is possible that none of these birds had an *E. apteryxii* infection or that infection was not present in the specific tissues tested. Given that the selection of tissue for initial post-mortem analysis likely influences what species are detected, it is possible that, on routine pathological examinations, standard sampling sites of the intestine in kiwi do not represent all *Eimeria* spp. infection sites. Species of poultry *Eimeria* are distinctly site-specific regarding the sections of the intestine that they inhabit, to the extent that the location of infection can be diagnostic to species-level (Johnson and Reid 1970; Jordan et al. 2019). Morgan et al. (2012) described a variation in spatial distribution of coccidia stages with differing morphologies within the intestine with a minimum of three different gametocyte morphologies, and at least one of these was only observed in the distal sections of the intestinal tract, which seems to suggest there may be a site-specific distribution for at least some species of kiwi *Eimeria* as well. To determine whether sampling site or size could be limiting factors in detection, further molecular analysis of sequential sections of fresh intestinal tissue, such as was carried out histopathologically by Morgan et al. (2013) and molecularly by Jarquín-Diaz et al. (2019), across a larger pool of samples would be valuable to try to determine both the site of infection of *E. apteryxii* as well as detect additional species that may cause enteric coccidiosis in kiwi. If species are highly site-specific within the intestine, isolation of DNA from individual species within the gastrointestinal tract may be possible via techniques such as microdissection of individual morphotypes from preserved tissue samples (Jones et al. 2004; Small et al. 2008; Post et al. 2009; Matsubayashi et al. 2012; Ilgūnas et al. 2019). The primers and probes developed in the present study were designed from sequences sourced from faecal DNA, which is challenging and more prone to errors given the high prevalence of mixed-species infection (Coker et al. 2023). Microdissection may also provide a more controlled approach to sourcing single-species DNA and assist in confirming the genetic identity of the remaining species of kiwi *Eimeria* that were unable to be molecularly described in this study.

A third, rarely observed *Eimeria* species that has recently been described, *E. koka* (Scheltema et al. 2025), was the only species molecularly detected in extraintestinal tissues, with the highest prevalence in renal samples, and no detectable infection in the intestine. Mature renal oocysts documented in kiwi by Morgan et al. (2013) were oval and ~ 17 × 12.4 µm in size and varied morphologically to those observed in the intestine. Accounting for the shrinkage caused by preservation, this matches well with the morphology of *E. koka*, which is oval and ~ 20.8 × 15.9 µm (Scheltema et al. 2025). While this species had not yet been identified when the original histopathological analysis was done by Morgan et al. (2013), the description in Scheltema et al. (2025) provides evidence from a single post-mortem case of extraintestinal infection by this species, and the presence of both sexual and asexual coccidial stages in renal tissues suggests that this species can complete reproduction in the kidneys.

The lack of detection of *E. koka* in the intestine is not definitive evidence that it does not cause intestinal infection. As discussed earlier, sampling site and size are likely to have influenced the detection of *Eimeria* species. However, the emerging pattern of infection observed in this small number of samples suggests that *E. koka* may be able to complete its lifecycle within the kidneys of kiwi. Infection in the liver, lungs, and spleen was also detected, in less than half of the examined birds, and occurred concurrently with renal infection in all except one case. Of these, no convincing sexual stages have been observed in any extraintestinal tissues, except the kidney. While it is possible that the lack of observation of sexual stages in these tissues may be an artefact of sampling, for example, sexual stages were not yet present when the bird died or were present in tissue that was not examined, this observation was also made independently in another study by Morgan et al. (2013). This suggests that possibly, the liver, lung and spleen are not the primary site of extraintestinal infection*.* Renal coccidiosis is relatively common in avifauna, and this would follow observations in other avian host species that have distinct renal and intestinal infecting species (for example, Gajadhar et al. 1983). It may be possible that *E. koka* is able to undertake one or more asexual reproductive stages in different extraintestinal organs before returning to the kidneys to complete its lifecycle, most likely transiting between organs via merozoites (Marquardt 1976; Morgan et al. 2013). Alternatively, the observed infective stages in the liver, spleen and lungs may represent a reproductive dead-end. The asexual stages of coccidia are generally far less site-specific than the sexual stages, and some studies have observed the development of early stages of merogony, which disappear without further development, in non-target cells (Marquardt 1981). If *E. koka* is renal-specific, it seems most likely that following development in the kidneys, oocysts are then excreted in urine or urates, via the ureters, as in other renal coccidia of birds (Page and Haddad 1995; Greenwald et al. 2024). The faecal pellet in kiwi (and other birds) contains a mix of urine, urates, and faeces, which makes it challenging to identify the endogenous source of excreted oocysts. Therefore, the analysis of tissues, either fresh or preserved, is the most reliable method of confirmation of renal infection (Leighton and Gajadhar 1986; Gajadhar and Leighton 1988; Greenwald et al. 2024). It would be useful to screen more tissues from post-mortem coccidiosis cases where enteric infection was absent to see if this renal infection pattern is more common in kiwi.

Given the presence of *E. koka* in multiple extraintestinal tissues in the majority of kiwi sampled, it is also possible that rather than representing a renal-specific disease, it is a species that causes disseminated coccidiosis. Dissemination is uncommon and is sometimes considered an aberrant form of coccidiosis (Ball et al. 1989; Helke et al. 2006), as most *Eimeria* spp. have a strong affinity for infection location (Pellérdy 1974; Novilla et al. 1981; Dubey et al. 2019) and complete their entire lifecycle within different cells or niches of a single organ (Marquardt 1976, 1981; Ball et al. 1989). Experimental suppression of the poultry immune system suggests that lowered immunity increases the opportunity for coccidia to disseminate (Ball et al. 1989). Immunocompromised or underdeveloped immunity in juveniles also has been suggested to influence the site of coccidial development in other animals. The most well-known example of this is *Eimeria reichenowi* and *Eimeria gruis* of cranes (*Grus* spp.), which develop in both the intestine and lungs and can spread systemically, forming granulomas in many different organs, a pattern of disease known as disseminated visceral coccidiosis (DVC) (Novilla et al. 1989; O’Brien et al. 2011). Similar infection patterns have also been observed in chamois (*Rupicapra rupicapra*) (Brunnert and Altman 1992) and echidna (*Tachyglossus aculeatus*) (Dubey and Hartley 1993; Debenham et al. 2012; Šlapeta et al. 2017).

While the majority of extraintestinal tissues were positive for *E. koka* via the qPCR probe assay, there were seven infected extraintestinal samples that tested positive for *Eimeria* species but were unable to be identified to species level. Therefore, to further verify the identification of a single species via qPCR in tissue samples, nine DNA samples from extraintestinal tissue were sequenced—of these, 7/9 were consistent with *E. koka*. Electropherograms of DNA sequenced from the remaining two tissues suggested the presence of *Eimeria* species; however, they could not be genetically identified to species level, and no coccidia stages were visually observed in suspect tissue from either case on histopathology. The lack of detection of *E. koka* in these tissues could be due to the presence of a second extraintestinal species of *Eimeria*; however, as most sequences collected from extraintestinal tissues did not suggest co-infection and the only coccidia stages observed were consistent with *E. koka*, there is currently no strong evidence for a second species. It is possible that the lack of DNA detection of *E. koka* in these tissues is a result of the very low infection levels observed within some of these tissues.

The sample quality, tissue preservation, and tissue type itself could also inhibit detection of the target species due to the quality of resulting DNA (Serth et al. 2000; Srinivasan et al. 2002; Gouveia et al. 2016; Dahn et al. 2022; Shi et al. 2023; Siuta et al. 2023). A portion of samples analysed in this study had low 260/230 ratios, likely indicative of the presence of sample contaminants, such as potential amplification inhibitors used in the fixing process, despite the use of an inhibitor-resistant mastermix for the probe-based qPCR assays. It is also likely that *Eimeria* spp. DNA sourced from FFPE tissues is of a low quality and/or fragmented and therefore more difficult to identify due to lower primer/probe binding to the target sequence. Thus, it is possible that the levels of coccidia infection reported here may underrepresent true levels of coccidia infection. Use of a house-keeping gene, such as PGK1, to monitor the levels of fragmentation due to the tissue preservation technique may be beneficial in future studies to validate this (Olias et al. 2014). To confirm the presence of a single species in extraintestinal tissues, a wider pool of case studies may need to be investigated and further molecular analysis of a range of fresh tissue samples is required to rule out the effects of preservation and potential sample contamination in the preparation of FFPE tissues.

Additionally, the lower fluorescence values attained in the Eimeria generic and EK-FAM assays suggest that further probe/assay optimisation may be required to improve the sensitivity of these two tests. This is not that surprising given the difficulty in isolating single-species DNA for the design of each of the species-specific probes. There is a higher possibility of error in probe design where more species are involved (*Eimeria* generic) or species differentiation is challenging (EK-FAM). It is possible that faecal samples used for EK-FAM design contained both *E. kiwii* and its morphologically identical counterpart, *E. paopaoii*. Samples visually identified as *E. kiwii* for the control panel may not have, in fact, included significant numbers of the target species, potentially resulting in a lower fluorescent signal for *E. kiwii*-positive samples. In addition, further development of the EK-FAM probe, to increase its Tm and improve target-specific binding, may be beneficial*.* Collection of more sequences for all species, particularly *E. kiwii*, would be beneficial to identify the presence and degree of sequence variation that could be reducing probe-binding and subsequent fluorescent signal.

Interestingly, in most of these severe cases of coccidiosis, both enteric and renal infection were involved. Although conclusions cannot be drawn from the small number of cases investigated in this study, it is possible that concurrent renal and enteric pathologies are more detrimental than one or the other on its own and certainly indicate more systemic parasitisation, possibly because of immunocompromise. The true pathogenicity of each kiwi *Eimeria* species may be difficult to ascertain without experimental infection studies, which are unlikely in a rare host species such as the kiwi. With further development of this assay, including validation of its sensitivity and specificity, it could be applied to faecal testing of a wider pool of live kiwi with differing severities and symptoms of coccidia infection to identify the presence of different *Eimeria* species. Collection of data from clinical cases may build a greater understanding of the relative pathogenicities of *E. kiwii*, *E. koka*, and others once assays are developed to detect them.

The evolutionary significance of extraintestinal infection is interesting to consider in the context of a species such as kiwi. In migratory Gruiformes, such as cranes and corncrakes (*Crex crex*) and in some waterfowl, it has been suggested that the ability of *Eimeria* parasites to complete their lifecycle extraintestinally may be advantageous as development of a chronic infection in their hosts, or having a much longer lifecycle, may allow these parasites to survive to re-infect their host and conspecifics in new locations, increasing their potential for geographic dispersal (Nation and Wobeser 1977; Novilla et al. 1989; Jeanes et al. 2013; Rideout et al. 2017; Serna et al. 2018), or to persist through harsh seasonal conditions that are not conducive to oocyst sporulation (Thomas et al. 1995). Kiwi are non-migratory, usually found alone or in pairs, and are dispersed within the natural environment (Taborsky and Taborsky 1992). They usually have multiple roost sites throughout their territory that they frequently move between and, despite their independent habit, have sometimes been observed to roost socially in groups of up to five birds, with roost switching suspected to have a social aspect (Jamieson et al. 2016). It may be possible that renal infection is advantageous for the parasite in this lifestyle as infection cycles may take longer, and oocysts may be spread further spatially to new roost sites. Considering individual kiwi usually have multiple roost sites throughout their territory (Jamieson et al. 2016), this may allow greater exposure of the parasite to other potential hosts, particularly if other individuals use the same roosts. Alternatively, considering that at least six species of *Eimeria* have been identified to date from kiwi, it is possible that where multiple *Eimeria* species infect a bird, changes in the distribution and ecological niche of one or other species may result from competitive exclusion, as has been documented in some other parasite species (Chappell 1969; Oliveira et al. 2022).

The individuals analysed in this study represent a small number of available post-mortem cases that were selected because they had severe extraintestinal coccidiosis and, as such, these cases are unlikely to be representative of the wider kiwi population. In contrast to kiwi, diagnosis of coccidiosis in poultry typically involves sacrifice of selected birds for the purposes of investigation and is likely to provide better representation of the study population. In addition, fresh tissues from sacrificed birds can be analysed immediately; however, because many kiwi have to be sent to specialists for post-mortem analysis, resulting in a delay in examination, autolysis of tissues is common and can influence the ability to detect tissue damage due to disease. A much larger pool of kiwi needs to be surveyed to determine typical patterns of disease; further samples from animals with mild clinical and/or non-clinical coccidiosis, particularly those with chronic low-level infection as seen in captive-reared birds, and of different ages need to be examined. This information will help to determine whether the infection of organs other than the renal or enteric tissues is an aberrant occurrence that only happens under certain physiological and environmental conditions or whether it is a normal part of the life cycle of this species of *Eimeria*. Disseminated coccidiosis is potentially more severe and leads to death of the host in other host species, including echidnas (Dubey and Hartley 1993) and cranes (Novilla et al. 1989); thus, the ability to identify the species involved and the conditions under which it disseminates is useful from a disease management perspective.

In this study, a molecular tool was developed to differentiate *Eimeria* species from faecal and tissue samples from infected juvenile kiwi. The application of this tool to preserved tissue samples demonstrates the likely distribution of two kiwi *Eimeria* species: one detected in the intestine (*E. kiwii*) and one in the kidneys (*E. koka*). This tool could be applied to determine the genetic identity of other *Eimeria* species infecting kiwi and their respective site-specificity, including the location of *E. apteryxii* infection. Further analysis of tissue and faecal samples with varying infection levels could be performed to further assess the application of this assay for the quantitative diagnosis of severity of coccidia infection in kiwi. With further development, the application of this molecular assay could be used to identify *Eimeria* species from the faeces of living birds and to test samples from clinical cases, potentially increasing understanding of species’ pathogenicity and individual species’ response to different anticoccidial treatments. This would be a major benefit for future management of disease, and prevention of species-specific anticoccidial resistance, in this threatened wildlife species.

## Supplementary Information

Below is the link to the electronic supplementary material.

ESM 1 (DOCX 3.17 MB)

ESM 2 (DOCX 17.4 KB)

## Data Availability

Sequence data were deposited in the NCBI GenBank database with the accession codes- PQ609358 (Eimeria apteryxii); PQ609359 (E. kiwii); PQ609361 (E. koka) and PQ609360 (Isospora sp. (ex. kiwi)).
