# Supplementary material for: Development of a qPCR molecular diagnostic assay for the detection of kiwi Eimeria species and its application to determine tissue-specificity of species causing coccidiosis in North Island brown kiwi (Apteryx mantelli)
Source: Parasitol Res. 2025 Jul 4;124(7):77. doi: 10.1007/s00436-025-08521-0 (PMC12226697; doi:10.1007/s00436-025-08521-0)
Supplement: Supplementary file 1 — (DOCX 3.17 MB) [file 436_2025_8521_MOESM1_ESM.docx]

**Supplementary File 1**

**Development of a qPCR molecular diagnostic assay for the detection of kiwi *Eimeria* species, and its application to determine tissue-specificity of species causing coccidiosis in North Island brown kiwi (*Apteryx mantelli*).**

**Authors: Emma Scheltema^1,a֍^, Kerri Morgan^2,b^, Stuart Hunter^2^, John Mackay^3,c^, Preet Singh^1,d^, Laryssa Howe^1,e^**

^1^ School of Veterinary Sciences, Massey University, Palmerston North, New Zealand

^2^ Wildbase, Massey University, Palmerston North, New Zealand

^3^ dnature diagnostics and research Ltd., Gisborne, New Zealand

Address correspondence to Emma Scheltema, e.scheltema@gmail.com


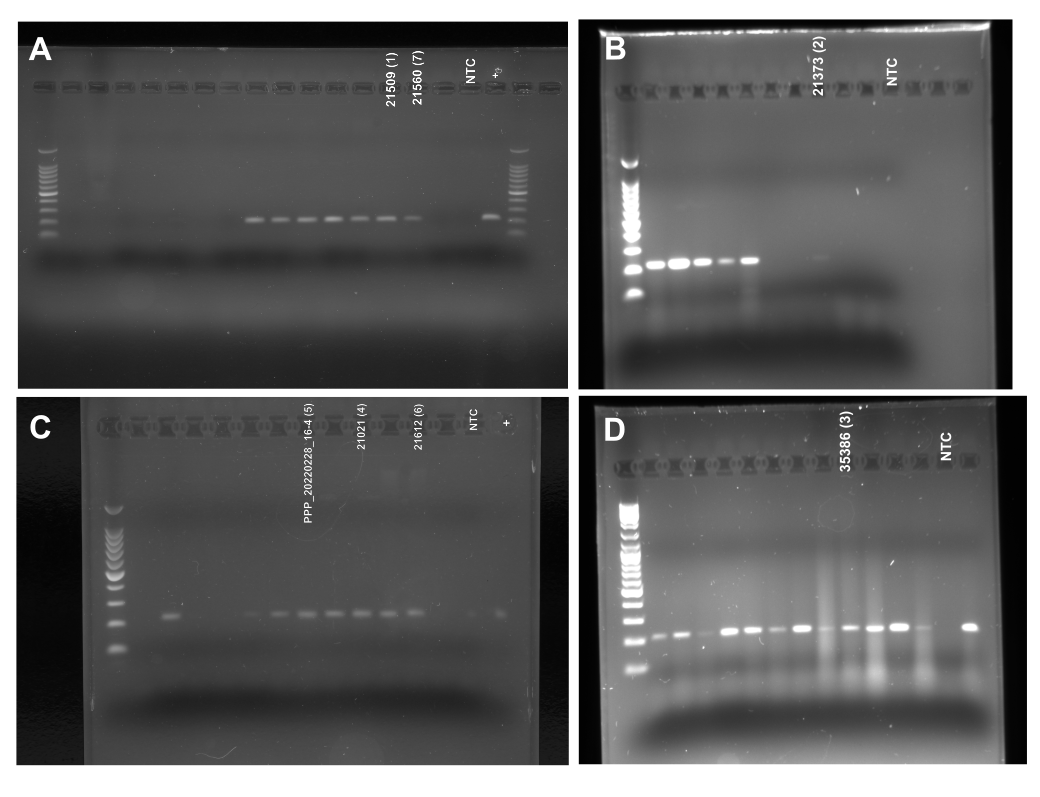


**Figure 1:** Representative *Eimeria* DNA samples (n=7) used for development of species detection qPCR, as visualized on 1.5% w/v agarose gel alongside 100bp ladder. Numbers in brackets refer to the same sample in **Fig.2**: (A) 21509 (1)– *Eimeria kiwii*, 21560 (7) – mixed: *Eimeria kiwii, Eimeria koka, Eimeria* spp, *Isospora*; (B) 21373 (2) – *Eimeria kiwii* and *E. apteryxii*; (C) PPP-20220303-16-4 (5) – *Eimeria paraurii* (suspected), 21021 (4) – *Eimeria koka*, 21612 (6) – *Isospora* spp. ex. kiwi; (D) 35386 (3) – infected hepatic tissue. No template controls are also indicated.
