# Supplementary material for: Development of a qPCR molecular diagnostic assay for the detection of kiwi Eimeria species and its application to determine tissue-specificity of species causing coccidiosis in North Island brown kiwi (Apteryx mantelli)
Source: Parasitol Res. 2025 Jul 4;124(7):77. doi: 10.1007/s00436-025-08521-0 (PMC12226697; doi:10.1007/s00436-025-08521-0)
Supplement: Supplementary file 2 — (DOCX 17.4 KB) [file 436_2025_8521_MOESM2_ESM.docx]

**Development of a qPCR molecular diagnostic assay for the detection of kiwi *Eimeria* species, and its application to determine tissue-specificity of species causing coccidiosis in North Island brown kiwi (*Apteryx mantelli*).**

**Authors: Emma Scheltema^1,a֍^, Kerri Morgan^2,b^, Stuart Hunter^2^, John Mackay^3,c^, Preet Singh^1,d^, Laryssa Howe^1,e^**

^1^ School of Veterinary Sciences, Massey University, Palmerston North, New Zealand

^2^ Wildbase, Massey University, Palmerston North, New Zealand

^3^ dnature diagnostics and research Ltd., Gisborne, New Zealand

Address correspondence to Emma Scheltema, e.scheltema@gmail.com

**Supplementary File 2**

**Table 1:** Demographic details of the ten North Island Brown kiwi (*Apteryx mantelli*) post-mortem cases (from 2012-2021) analysed in this study.

| **Accession #** | **Year** | **Sex** | **Location** | **Captive, creche or wild** | **Age (at PM)** | **Cause of death (in order of significance)** |
| --- | --- | --- | --- | --- | --- | --- |
| **49058** | 2012 | Male | Opouahi Kiwi Creche, Lake Opouahi Scenic Reserve, Hawkes Bay | Creche | ~ 8 days old | Severe enteric and renal coccidiosis* |
| **50621** | 2014 | Male | Opouahi Kiwi Creche, Lake Opouahi Scenic Reserve, Hawkes Bay | Creche | ~32 days old | Emaciation, enteric and renal coccidiosis* |
| **51484** | 2014 | Male | Opouahi Kiwi Creche, Lake Opouahi Scenic Reserve, Hawkes Bay | Creche | ~32 days old | Emaciation, severe enteric and renal coccidiosis |
| **51506**** | 2014 | Female | Opouahi Kiwi Creche, Lake Opouahi Scenic Reserve, Hawkes Bay | Creche | ~47 days | Enteric and renal coccidiosis*, secondary emaciation with lymphoid atrophy (thymus and spleen) |
| **56082** | 2018 | Male | Taumaranui | Wild | Juvenile | Head trauma, haemorrhage, mild coccidiosis |
| **58596** | 2020 | Male | National Kiwi Hatchery Aotearoa, Rotorua | Captive-reared | 10.5 weeks | Acute necrotising pancreatitis, severe hepatic lipidosis |
| **58631** | 2020 | Male | Rotokare Scenic Reserve, Eltham, Taranaki | Creche | 7 months | Poor body condition, enteric and hepatic coccidiosis* |
| **58704** | 2020 | Male | Whangarei | Captive-reared | 6 weeks | Emaciation, ventricular nematodiasis, enteric coccidiosis* |
| **59312** | 2020 | Male | National Kiwi Hatchery Aotearoa, Rotorua | Captive-reared | ~6 weeks | Severe verminous ventriculitis |
| **59468** | 2021 | Male | Pukaha National Wildlife Park, Eketahuna | Captive-reared | ~ 4 weeks | Severe ulcerative and verminous ventriculitis, secondary malabsorption and bacteria enteritis |

* indicates coccidiosis was identified as a primary cause of death
** case was partially analysed in a previous study (Scheltema, 2025**)**
